# Silver(i) pyridylphosphonates – synthesis, structure, stability and light-insensitivity investigation†

**DOI:** 10.1039/c8ra10136a

**Published:** 2019-01-11

**Authors:** Michaela Rendošová, Róbert Gyepes, Miroslav Almáši, Ingrida Bártová, Zuzana Vargová

**Affiliations:** Department of Inorganic Chemistry, Faculty of Science, P. J. Šafárik University Moyzesova 11 041 54 Košice Slovak Republic zuzana.vargova@upjs.sk; Department of Inorganic Chemistry, Faculty of Science, Charles University Hlavova 2030 128 00 Praha Czech Republic

## Abstract

Two silver(i) complexes, {[Ag_7_(2-pypo)_3_(NO_3_)]}_*n*_ (1) and [Ag(3-pypoH)(3-pypoH_2_)] (2) (pypoH_2_ – pyridylphosphonic acid) were prepared and characterized by relevant methods in the solid state (elemental, spectral, thermal and structural analysis). Typical argentophilic interactions were observed in complex 1. The synthesized Ag(i) complexes were tested toward reduction to silver(0) using short wavelength UV-radiation and they revealed remarkable light-insensitivity in comparison to silver nitrate. SEM experiments were performed to observe the reduction of silver after the UV-light exposure of the samples. Light-insensitivity with the examined thermal stability and insolubility of the new complexes demonstrates their high potential to be used as light-insensitive materials in medical devices.

## Abstract 2

Two silver(i) complexes, {[Ag_7_(2-pypo)_3_(NO_3_)]}*_n_* (1) and [Ag(3-pypoH)(3-pypoH_2_)] (2) (pypoH_2_ – pyridylphosphonic acid) were prepared and characterized by relevant methods in the solid state and were tested toward reduction to silver(0).

## Graphical abstract

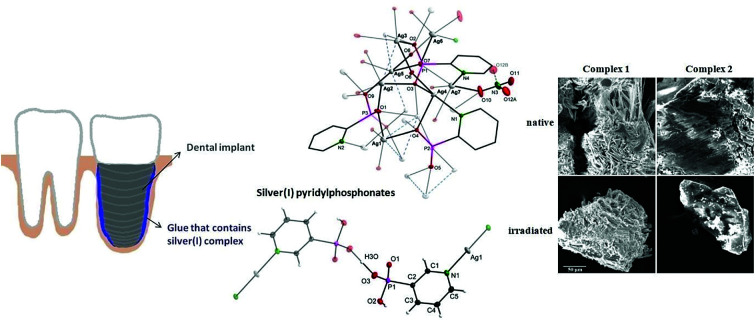

## Introduction

The chemistry of metal phosphonates has attracted attention in the last few years. Great effort has been devoted to the syntheses of metal complexes and extended networks utilizing pyridylphosphonic acids as promising candidates for functional materials^1^ with potential applications in catalysis, proton conductivity, ion exchange and magnetic materials, *etc.*^2^ Since phosphonic acids can be readily deprotonated, they form anionic ligands that can coordinate to metal centres and compensate for charge^3^ having several coordination modes. Another advantage of these ligands is their ability to induce polymerization as a result of the strong interaction of the phosphonate group with the metal ion.^3^

In the field of biomedical devices, clinical practice has shown that systemic antibiotics are not able to provide sufficient treatment for implant-associated infections.^4^ Upon increasing bacterial resistance against current antibiotics the development of new infection-preventing strategies is very important and research on silver and its compounds has regained interest.^5^ While the number of implanted medical devices is rapidly increasing, the infections associated with biomaterials still represent a significant challenge.^6^ Bacterial adhesion to implants and biofilm formation causes serious implant-related infections. For example, adhesion of bacteria to dental implant components may lead to the formation of a biofilm that could be responsible for dental diseases like dental caries, periodontal diseases and peri-implantitis.^7^ One of the possibilities how to prevent the infections is the introduction of an antimicrobial substance, for example, into the glue used in indwelling medical devices introduction or in surface treatment of the devices to prevent pathogenic cell adhesion and subsequent biofilm formation.^4^ Therefore silver ions which exhibit low cytotoxicity to mammalian cells and broad-spectrum antimicrobial activity have gained attention in a wide range of medicinal applications.^5^ Experiments with silver salts have led to unsatisfactory results. On the other hand, silver coordination polymeric compounds are promising candidates for surface modification as they exhibit (i) high structural stability during sterilization at 100 °C, (ii) little decomposition upon light exposure, (iii) poor solubility in aqueous media, and (iv) antimicrobial activity.^4,7^ Pure organic compounds cannot fulfil these specific needs, but some water-insoluble metal complexes (*e.g.* silver(i) compounds) can.^4^ For this reason, it is important to study new metal-based compounds that will meet these requirements.^4^ In this work we present results of our investigation dealing with the synthesis, spectroscopic, structural and photophysical characterization of the new silver(i) pyridylphosphonates {[Ag_7_(2-pypo)_3_(NO_3_)]}_*n*_ (1) and [Ag(3-pypoH)(3-pypoH_2_)] (2) as promising materials for implant applications.

## Results and discussion

### Syntheses of complexes

Complex 1 was synthesized from aqueous solution of 2-pyridylphosphonic acid and silver nitrate at 1 : 1 molar ratio. After slow evaporation (one week) at room temperature in the dark, small colourless crystals of compound 1 were formed. The 1 : 1 molar-ratio reaction of 3-pyridylphosphonic acid and silver nitrate in a mixed solvent H_2_O : DMSO = 5 : 1 (DMSO = dimethyl sulfoxide) yielded complex 2. Under the same conditions of evaporation, grey acicular crystals were formed after three weeks. The compounds were stored in the absence of light, they are air-stable and insoluble in water and organic polar solvents.

### Solid-state study

#### Crystal structure

**Fig. 1:**
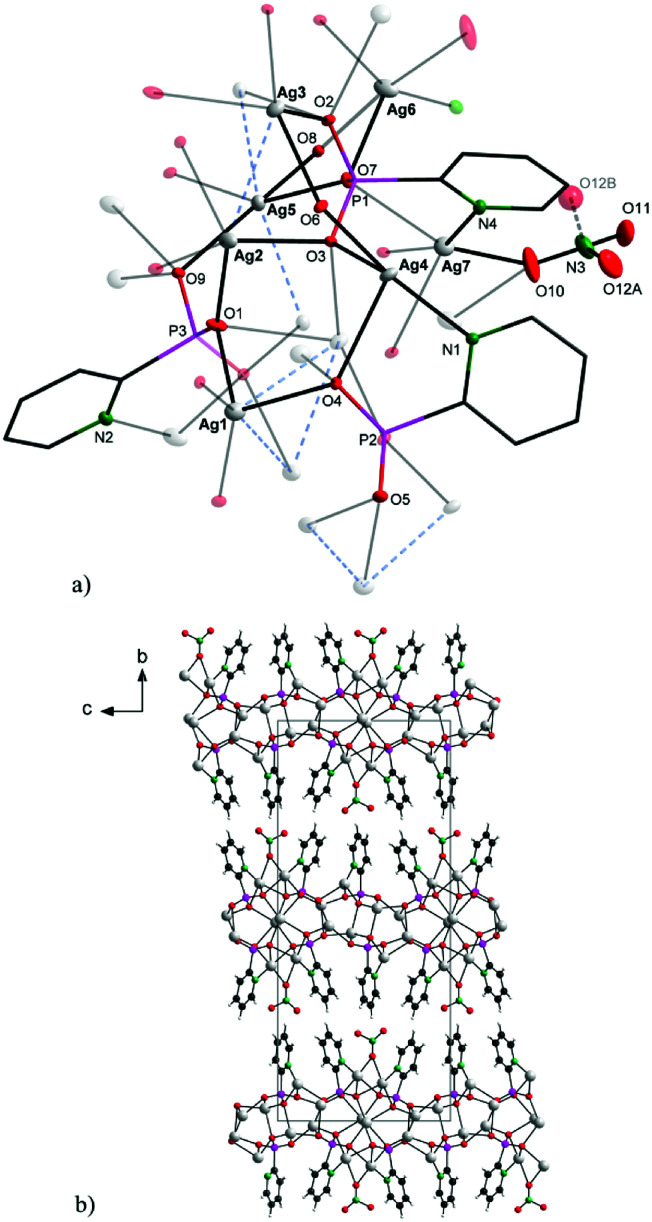
Solid-state structure of the symmetrically independent part (a; upper) and packing diagram viewed along [1 0 0] (b; bottom) for 1. For clarity, labels in the upper picture are given for the most important atoms; atom O12B is omitted on the bottom.

Complex 1 crystallizes in a monoclinic lattice with space group *P*2_1_/*n*. The asymmetric part of the unit cell incorporates three pyridylphosphonato anions 2-pypo, seven silver atoms and a single nitrato ligand, which has one of its oxygen atoms disordered over two positions. The central atoms Ag1–Ag5 have all distorted tetrahedral geometry, while the coordination environment of both Ag6 and Ag7 is a distorted square pyramid. A different coordination environment around silver centres Ag4, Ag6 and Ag7 is generated by including the pyridine nitrogen in the coordination. Additionally, Ag6 and Ag7 are both involved in coordinating to the nitrato group through O10. The O12 atom of the nitrato group is disordered over two positions (labelled as O12A and O12B) and was refined with the sum of both positions equalling one. The refined occupancy for O12A was 0.633(8); O12B could only be refined isotropically and is drawn semi-transparently in Fig. 1a.

As shown in Fig. 1, the connectivity is quite complicated: the 2-pypo ligand acts as multi-bridging ligand – all of its three oxygen atoms are involved in coordination with silver ions; in addition, the 2-pypo ligand acts as a *N*,*O*-chelating ligand, creating a two-dimensional layered structure with layers packed in the *ac* plane (Fig. 1b).

The P–O interatomic distances are approximately halfway between the P–OH bond and the formal double bond P=O,^8^ pointing to the negative charge delocalization over the P–O bonds.^9^ The nitrato ligand is connected with Ag7 and Ag6 having the Ag6–O10 distance 2.658(4) Å and Ag7–O10 2.471(3) Å. The other Ag–O (oxygen atoms of phosphonate groups) distances are in the range 2.234(2)–2.661(3) Å, while the Ag–N distances are 2.212(3) Å, 2.237(3) Å and 2.284(3) Å. These distances together with other bond lengths and angles (see Table S1 in ESI†) are in good accordance with previously published values for other silver complex {[Ag_3_(triflate)_2_(2-Hpypo)]}_*n*_.^3^ Moreover, close Ag–Ag contacts had been observed with the metal–metal distance of 2.9298(5)–3.3787(4) Å (Fig. 1a, blue dashed bonds). These contacts are shorter than the sum of their van der Waals radii (3.44 Å), indicating the presence of significant argentophilic interactions.^10,11^

**Fig. 2:**
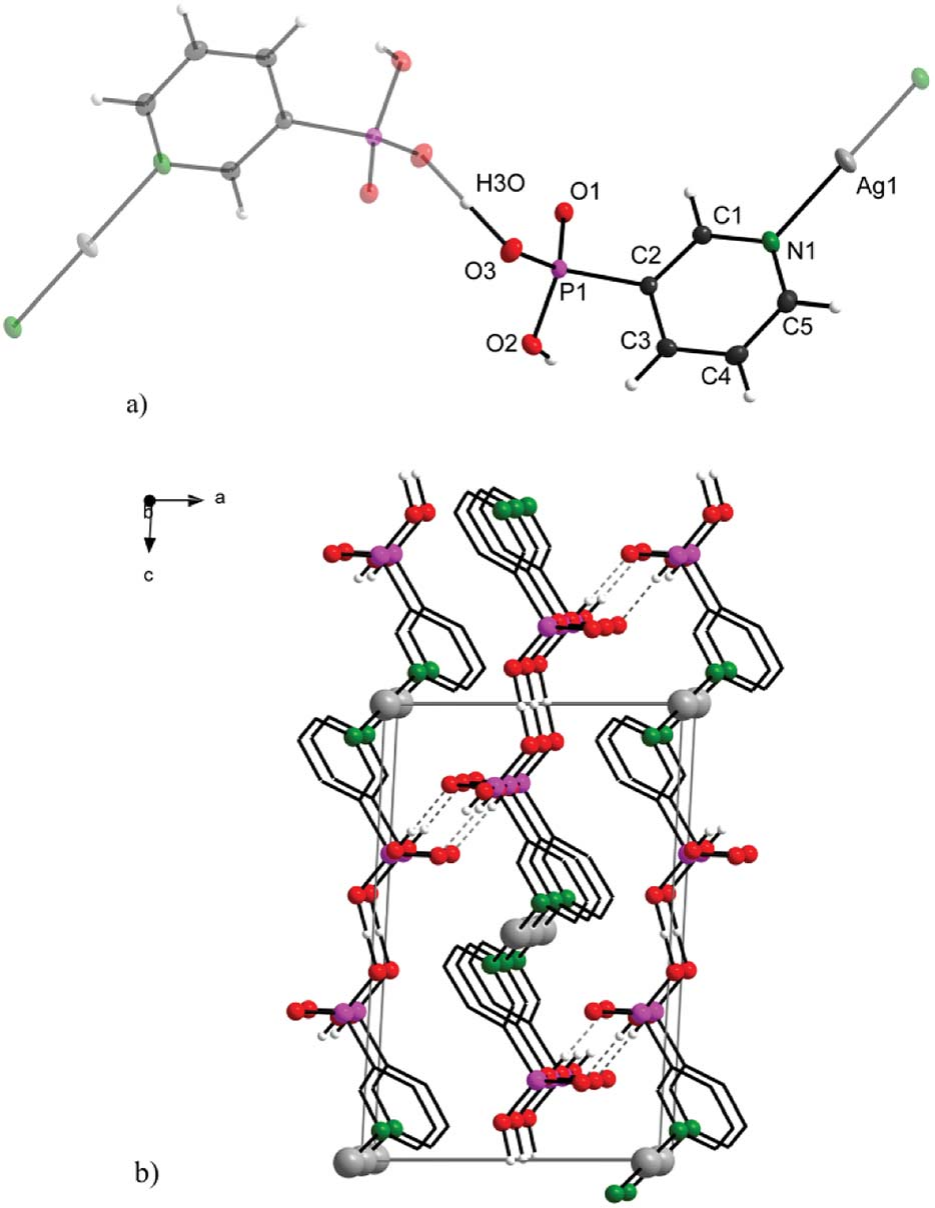
Structure motif (a; upper) and solid-state packing viewed along [0 1 0] (b; bottom) for 2.

Complex 2 crystallizes in a monoclinic lattice with space group *P*2_1_/*n*. The 3-pypo ligand is coordinated to the silver central atom through its pyridine nitrogen. The Ag–N distance is 2.1219(15) Å, which is even shorter than its analogue in 1. Other bond lengths and angles are in good accordance with previously published values for 3-pyridylphosphonic acid^8^ and other metal complexes with 3-pypo ligand^12^ and are listed in Table S2 in ESI.† The coordination number of Ag(i) in the complex is two, having a linear arrangement of the nitrogen donor atoms. The 3D arrangement is formed through O3–H3O–O3 hydrogen bonding interactions, where the hydrogen is located in the middle of O–O distance (Fig. 2). Apart from this intermolecular interaction, the structure is stabilized also by O2–H2⋯O1 hydrogen bonds (Fig. 2).

Additional π–π stacking interactions are found between the pyridyl rings of the 3-pyridylphosphonato ligands, with an intercentroid distance of 3.7926(11) Å and interplanar spacing 3.4074(7) Å. The ring normal and the vector between the ring centroids form an angle of 26.0°. These values are commonly observed values in complexes with aromatic nitrogen heterocycles.^13^ The chemical composition of 1 and 2 was in good agreement with results of elemental analyses.

#### IR spectra

The IR spectra of 2-pyridylphosphonic acid, 3-pyridylphosphonic acid, complexes 1 and 2 measured at room temperature are shown in Fig. S1 in ESI.† Generally, the C–H pyridine stretching vibrations of heterocyclic compounds are observed in the region of 3110–3000 cm^−1^.^14^ In the spectra of free acids, these absorption bands are observed at 3108 and 3082 cm^−1^ for 2-pypoH_2_ and 3118 and 3084 cm^−1^ for 3-pypoH_2_. In the case of coordinated pyridylphosphonic acids, corresponding vibrations are slightly shifted to the lower values of the wavenumber (Table 1). According to the literature, the characteristic C–C and C–N pyridine ring stretching vibrations are located in the ranges of 1620–1595 and 1460–1410 cm^−1^ (ref. 15) and these vibrations are observed in the spectra of 1 and 2 in the range of 1598–1417 cm^−1^ (Table 1). In the spectra of pyridylphosphonic acids broad bands between 3550–3150 cm^−1^ are observed which belong to O–H stretching vibrations, while in the spectrum of 1 no bands in the mentioned region were observed. That fact corresponds with the coordination mode of 2-pyridylphosphonate through all the three oxygen atoms of phosphonate group. Interestingly, in the spectrum of 2 the O–H stretching vibrations are not observed as was expected. This fact may be related to the presence of specific hydrogen bond interactions described above. In all spectra, several bands between 1250 and 950 cm^−1^ correspond to the characteristic stretching modes of the PO_3_ group.^15^ The wavenumber values are shifted from 1157, 1091 and 1030 cm^−1^ in the case of 2-pypoH_2_ to 1164, 1154 and 1031 cm^−1^ in the case of complex 1 due to its coordination to silver ions through all three oxygen atoms. On the other side, there is no coordination of PO_3_ group with silver ion in complex 2 and the shifting of these bands is also observed. The PO_3_ deformation vibrations are generally found in the region 555–415 cm^−1^.^16^ In the spectrum of 2-pypoH_2_ corresponding vibrations are observed at 553, 513 and 439 cm^−1^, while mentioned vibrations are shifted to higher wavenumber values at 570, 528 and 460 cm^−1^ in the spectrum of 1. Similarly, the vibrations are shifted from 535, 511 and 443 cm^−1^ in spectrum of 3-pypoH_2_ to 555, 485 and 450 cm^−1^ in spectrum of 2. The stretching C–P band is observed in all spectra in the range of 948–923 cm^−1^. IR spectrum of complex 1 confirms the nitrato ligand presence due to the observed N–O vibration at 1316 cm^−1^.

**Table 1:** The IR spectral data assignments for complexes 1 and 2 and their appropriate ligands 2- and 3-pyridylphosphonic acids

	2-pypoH_2_	Complex 1	3-pypoH_2_	Complex 2
*ν*(O–H)	3480–3300	—	3550–3180	—
*ν*(CH)	3108, 3082	3054, 3007	3118, 3084	3110, 3078
*ν*(C–C), *ν*(C–N)	1608, 1520, 1443, 1389	1583, 1562, 1459, 1420	1626, 1548, 1460, 1368	1598, 1566, 1478, 1417
*ν*(PO_3_)	1157,1091, 1030	1164, 1154, 1031	1178, 1112, 1034	1196, 1159, 1118
*ν*(C–P)	939	948	932	923
*ν*(NO_3_^−^)	—	1316	—	—
*δ*(PO_3_)	553, 513, 439	570, 528, 460	535, 511, 443	555, 485, 450
γ(C–H)	765, 717	760, 726	727, 682	748, 703

In summary, observed band shifting in the spectra of complexes may be related to the metal ion coordination as well as to the intermolecular interactions.

#### Thermogravimetric analysis

**Fig. 3:**
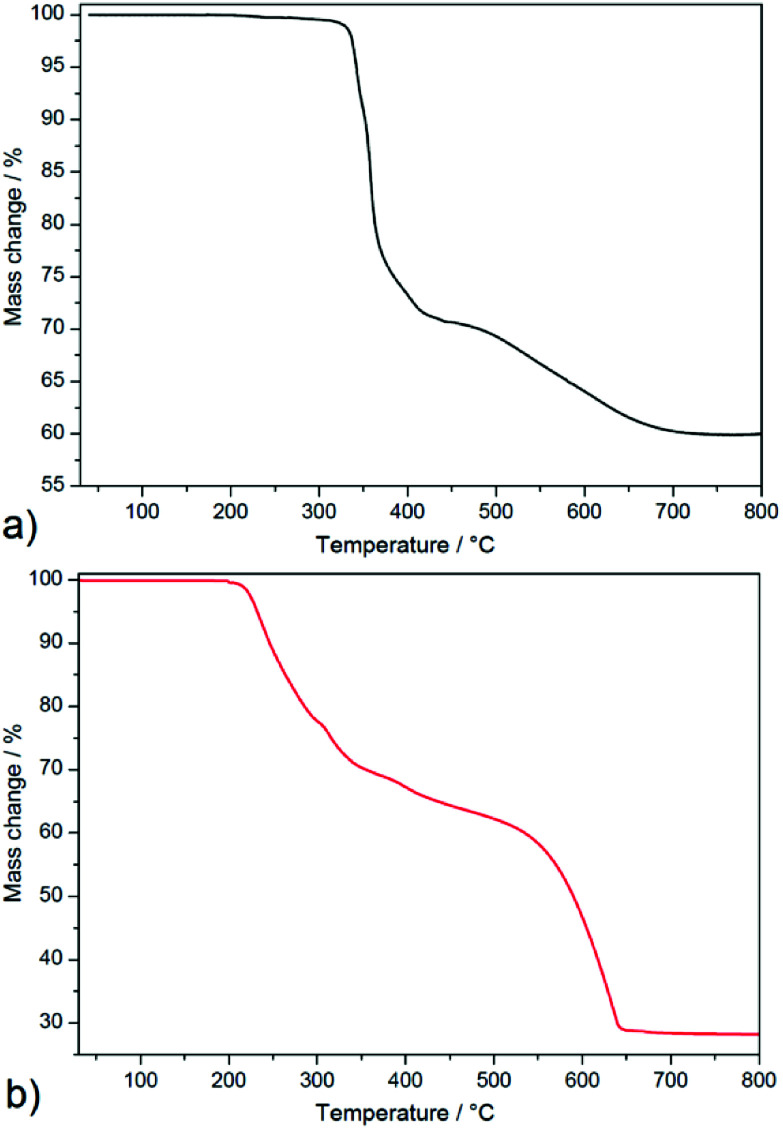
Thermogravimetric curves of 1 (a) and 2 (b) measured in temperature range 30–800 °C and air atmosphere.

To estimate the thermal stability of compound 1 and 2, thermogravimetric analysis was carried out under air atmosphere and obtained TG curves are shown in Fig. 3. Due to complicated structure of 1, the observed decomposition steps on TG curve could not be attributed to individual building components of the compound and therefore thermal decomposition is complicated. We can only summarize, that thermal decomposition of organic/inorganic part of complex 1 (2-pypo and NO_3_^−^ ions) takes place in the temperature range of 325–730 °C (see Fig. 3a) with observed mass loss 40.1%, which is in good agreement with calculated value 41.4%. The final decomposition product was elemental silver with residual mass 59.9% (clcd. residual mass 58.6%). Observed thermal stability of 1 is higher comparing to ternary complex which consists of silver(i), 2-Hpypo and triflate (CF_3_SO_3_^−^) ions in which crystal structure silver ions are connected into the zig-zag chains.^3^ This compound melts at 176 °C and decomposes in the temperature range of 226–384 °C. Higher thermal stability of 1 could be attributed to stronger Ag–Ag interactions which are arranged into the 1D polymeric tubules and higher crystal packing of building blocks compared to {[Ag_3_(triflate)_2_(2-Hpypo)]}_*n*_.

Complex 2 (see Fig. 3b) is thermally stable up to 200 °C and thermal decomposition of the organic part occurs in four overlapping decomposition steps. The first three decomposition steps appeared in the temperature range of 200–440 °C in which mass loss of 37.2% was observed and correspond to the thermal decomposition of one 3-pypoH_2_ molecule (clcd. mass loss 37.4%). The second deprotonated ligand molecule is evolved in the temperature range of 440–650 °C, with mass loss of 36.2% (clcd. mass loss 37.2%). The final product of the thermal decomposition was also silver with residual mass 26.6% (clcd. residual mass 25.4%). Comparison of thermal stability could be performed only for a few compounds, such as {[Cd_2_(3-pypoH)_4_]}_*n*_·2*n*DMSO^15^ and {[Sn_2_(3-pypo)_2_]}_*n*_,^1^ which display thermal stability 360 and 325 °C, respectively^1,15^ (for compound {[Cd_2_(3-pypoH)_4_]}_*n*_·2*n*DMSO after removal of the solvent molecules). Higher stability of described compounds could be explained by the polymeric nature of their frameworks compared to the molecular structure of compound 2.

In summary, complex 1 displayed higher thermal stability compared to 2. Its higher thermal stability compared to complex 2 could be attributed to the higher crystal packing and complicated covalent bonding formations between building blocks in the crystal structure of 1. Moreover, the conjunction between Ag–Ag interactions helps to stabilize the polymeric network, which resulted in higher thermal robustness of 1.

#### UV-light stability studies

**Fig. 4:**
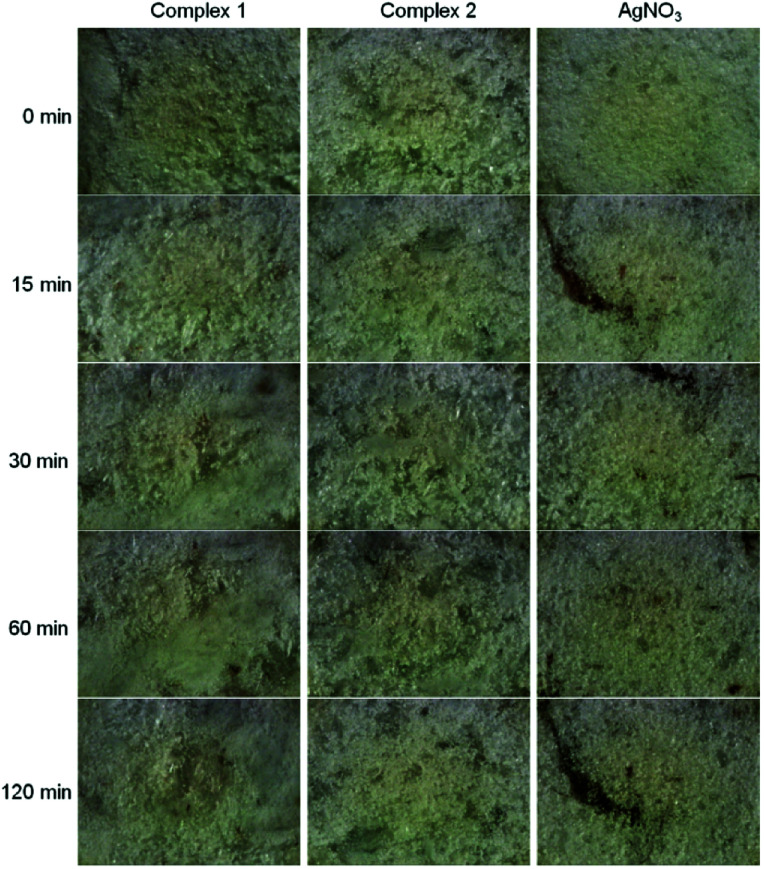
Light-insensitivity study of new silver(i) pyridylphosphonates toward UV-radiation at 254 nm in comparison to silver nitrate.

The results of stability testing of Ag(i) compounds toward reduction to silver(0) by short wavelength UV-radiation is shown in Fig. 4. Complexes 1 and 2 show good stability for a prolonged period of time in comparison with the control photosensitive substance AgNO_3_. After 15 minutes of radiation, dark spots appeared on the sample of AgNO_3_ that gradually darkened within 120 minute of exposure. Moreover, dark spots were visible with the naked eye. The polymeric silver compounds 1 and 2 exhibit remarkable UV-light insensitivity. After 120 min of exposure, the number of spots was lower and spots were smaller and lighter in comparison with AgNO_3_. However complex 2 exhibits higher UV-light stability in comparison with complex 1; a number of the dark spots appeared after 30 minutes of the UV-light exposure in the case of complex 1 and after 120 minutes of the exposure in the case of 2. These differences toward UV-light could be related to the different short covalent bonds between silver(i) and donor atoms of the ligand and the bond energies.^17^

The light-insensitive characteristics of silver(i) compounds as well as their thermal stability and poor solubility are important for their potential applications in medical devices as additives to curable photopolymers in dental implants.^4,17^ Practically, for the intention of desired application of synthesized compounds only minutes of light stability are needed.^4^

#### SEM observations

**Fig. 5:**
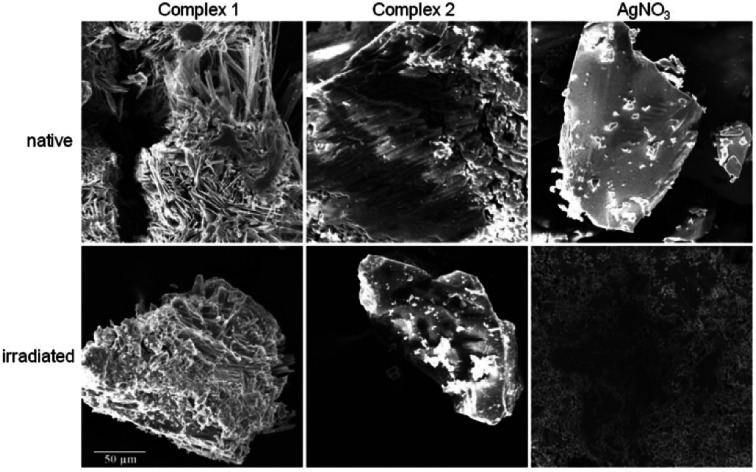
SEM images of native and irradiated silver(i) complexes and silver nitrate at voltage of 20 kV. The scale applies to all images.

SEM experiments were performed to observe the reduction of silver after the UV-light exposure of the samples. The samples were not conductive and displayed the charging effect during the SEM experiment. SEM images of complexes 1 and 2 in native state and after irradiation are without significant differences. On the other hand, the images of native and irradiated silver nitrate are significantly different due to the observation of the colour change and minor charging effect. Dark spots are observed on the surface of the sample of AgNO_3_ after UV-light exposure (Fig. 5). This experimental fact could be related to the density changes; dark spots represent elemental silver with higher density resulted after the irradiation of AgNO_3_ (with lower density).

## Conclusions

Two silver(i) pyridylphosphonates were prepared and fully characterized by IR, CHN, TG and X-ray analysis. Typical argentophilic interactions were observed in complex 1. The IR spectra of both complexes are in a good correlation with structural observations. As was demonstrated by thermal analysis, complexes are thermally stable up to 200 °C, while complex 1 exhibits even higher thermal stability than complex 2. The synthesized Ag(i) complexes have demonstrated remarkable light-insensitivity toward UV-radiation in comparison to silver nitrate. SEM observations revealed dark spots on the most UV-sensitive sample, silver nitrate, that may represent reduced elemental silver on the surface of the irradiated sample.

Thermal stability, light-insensitivity and poor solubility are important properties of potential materials for dental implants and indwelling medical devices. A high thermal stability of used materials is required for equipment sterilization and water insolubility is needed for no leaching them out from the surface of the indwelling devices. In conclusion, new silver(i) pyridylphosphonates fulfil these requirements for their potential applications in medical devices and demonstrate high potential to be used as light-insensitive materials and therefore they are suitable candidates for further biocompatibility studies.

## Experimental

### Materials

Silver(i) nitrate was purchased from Lachema (Czech Republic), dimethyl sulfoxide (DMSO), palladium(ii) acetate (Pd(OAc)_2_) and 3-bromopyridine from Sigma-Aldrich Chemie, 2-bromopyridine, diisopropyl phosphite and *N*,*N*-diisopropylethylamine from Fluka, 1,1′-bis(diphenylphosphino)ferrocene (dppf) from Alfa Aesar, bromotrimethylsilane from Acros, dichloromethane, methanol and acetonitrile from VWR Chemicals.

### Synthesis of 2- and 3-pyridylphosphonic acids

Acids were prepared according to Belabassi:^18^ diisopropyl phosphite (4.8 mmol, 1.2 equiv.), 2- or 3-bromopyridine (4.0 mmol, 1.0 equiv.), *N*,*N*-diisopropylethylamine (5.2 mmol, 0.9 mL, 1.3 equiv.), Pd(OAc)_2_ (0.04 mmol, 1 mol%), and 1,1′-bis(diphenylphosphino)ferrocene (dppf) (0.044 mmol, 1.1 mol%) were added to acetonitrile (dry, 15 mL) and the reaction mixture was then heated at reflux for 24 h under argon atmosphere. After reaction, the solution was concentrated under reduced pressure (LC separation, TLC detection). The resulting oil was dissolved in CH_2_Cl_2_ and treated with bromotrimethylsilane (2.2 equiv.) at room temperature under N_2_. When silylation was complete (24–48 h, monitored by ^31^P NMR), the solvent was removed *in vacuo* and MeOH was added. The phosphonic acids were obtained as solids either directly, or by precipitation from water.

#### 2-Pyridylphosphonic acid

^1^H NMR (D_2_O): *δ* 8.00–8.02 (CH, 1H, t, ^4^*J*_HH_ = Hz); 8,17–8.19 (CH, 1H, t ^3^*J*_HH_ = Hz); 8.53–8.56 (CH, 1H, t, ^2^*J*_HH_ = Hz); 8.69–8.70 (CH, 1H, d, ^5^*J*_HH_ = Hz) ppm; ^31^P NMR: *δ* 0.19 (s) ppm; MS: (−) 157.6 (−2H^+^).

#### 3-Pyridylphosphonic acid

^1^H NMR (D_2_O): *δ* 10.35–10.60 (CH, 1H, t, ^4^*J*_HH_ = Hz); 11.21–11.29 (CH, 1H, t ^3^*J*_HH_ = Hz); 11.36–11.39 (CH, 1H, t, ^2^*J*_HH_ = Hz) ppm; ^31^P NMR: *δ* 5.97 (s) ppm; MS: (−) 157.6 (−2H^+^).

### Synthesis of complex {[Ag_7_(2-pypo)_3_(NO_3_)]}_*n*_ (1)

Silver(i) nitrate (100 mg, 0.59 mmol) was dissolved in water (5 mL) and added dropwise to the 5 mL of 2-pyridylphosphonic acid aqueous solution (93.6 mg; 0.59 mmol). The reaction mixture was stirred constantly for 10 min and then allowed to stand at room temperature. Crystals formed by slow evaporation (after five days) were filtered off, washed with diethyl ether and dried under vacuum for 24 h. Yield: 27%. Elemental analysis – found: C, 13.80; H, 0.76; N 4.51. Calc. for C_15_H_12_N_4_O_12_P_3_Ag_7_: C, 13.98; H, 0.94; N, 4.35%.

### Synthesis of complex [Ag(3-pypoH)(3-pypoH_2_)] (2)

Silver(i) nitrate (100 mg, 0.59 mmol) was dissolved in water (5 mL) and added dropwise to the 18 mL of 3-pyridylphosphonic acid H_2_O : DMSO (5 : 1 v/v) solution (93.6 mg; 0.59 mmol). The reaction mixture was stirred constantly for 10 min and then allowed to stand at room temperature. Crystals formed by slow evaporation (after three weeks) were filtered off, washed with diethyl ether and dried under vacuum for 24 h. Yield: 58%. Elemental analysis – found: C, 28.16; H, 2.45; N, 6.61. Calc. for C_10_H_11_N_2_O_6_P_2_Ag: C, 28.26; H, 2.61; N, 6.59%.

### Physical measurements

Diffraction data for 1 were measured on a Bruker D8 Venture Kappa Duo diffractometer equipped with a PHOTON 100 detector and IμS 3.0 microfocus sources; data for 2 were obtained on a Nonius Kappa CCD diffractometer equipped with a Bruker APEX II detector and graphite monochromator. For both measurements, Mo Kα (*λ* = 0.71073 Å) was used as the primary radiation. The phase problem was solved by intrinsic phasing and structure models were refined against F^2^ using the SHELX program suite.^19^ All non-hydrogen atoms (excepting O12B in 1) were refined anisotropically. All hydrogen atoms were refined isotropically; those on aromatic rings were included in idealized positions. Important crystallographic data are listed in Table S3 in ESI.† Structure figures were drawn using the DIAMOND software.^20^

Infrared spectra were recorded on Avatar FT-IR 6700 spectrometer from 4000 to 400 cm^−1^ using ATR (attenuated total reflectance) technique.

Elemental analysis was performed with a CHNOS Elemental Analyzer Vario MICRO from Elementar Analysensysteme GmbH.

Thermal behaviour of compounds 1 and 2 was studied by thermogravimetry (TG) using TA instrument TGA Q-500 in atmosphere of air. Samples were heated with heating rate 10 °C min^−1^ in temperature range from 30 to 800 °C and air flow rate 60 cm^3^ min^−1^. Before thermal measurements, gentle grinding of the sample and careful packing into the platinum crucibles were performed. The mass of samples used in the analyses was within 10–15 mg. Obtained thermoanalytical curves were analysed using Origin computational program (version 6.1052, Origin Lab Northampton, MA, USA).

Light-sensitivity of synthesized Ag(i) pyridylphosphonates was studied at 298 K using an ultraviolet germicidal lamp PROLUX G with a hot cathode (30 W, 254 nm). Complexes were gently ground into a powder and placed on a microscope slide. The radiation source was positioned 10 cm from the samples. Silver nitrate was used as a control substance to access the light stability of silver(i) complexes. An optical microscope with a 10 times microscope objective lens was used to observe the changes and digital pictures of the random part of the samples were taken in intervals 0, 15, 30, 60 and 120 min of radiation exposure time using a digital ocular camera (Dino Eye, 5 Mpx). Images were used without any further contrast or brightness editing. SEM images were taken with Scanning Electron Microscope VEGA (TESCAN) at 20 kV using secondary electron detector and back scatter electron detector.

## Conflicts of interest

There are no conflicts to declare.

## Supplementary Material

RA-009-C8RA10136A-s001

RA-009-C8RA10136A-s002
