# Supplementary material for: Silver(i) pyridylphosphonates – synthesis, structure, stability and light-insensitivity investigation
Source: RSC Adv. 2019 Jan 11;9(3):1570–5. doi: 10.1039/c8ra10136a (PMC9059710; doi:10.1039/c8ra10136a)
Supplement: RA-009-C8RA10136A-s001 [file RA-009-C8RA10136A-s001.pdf]

Electronic Supplementary Material (ESI) for RSC Advances.
This journal is © The Royal Society of Chemistry 2018

Electronic Supplementary material (ESI)

Silver(I) pyridylphosphonates – synthesis, structure, stability and light-insensitivity investigation

M. Rendošová, [a] R. Gyepes, [b] M. Almáši, [a] I. Bártová [a] and Z. Vargová *[a]

[a]*Department of Inorganic Chemistry, Faculty of Science, P. J. Šafárik University, Moyzesova 11, 041 54 Košice, Slovak Republic. E-mail: zuzana.vargova@upjs.sk*

[b]*Department of Inorganic Chemistry, Faculty of Science, Charles University, Hlavova 2030, 128 00 Praha, Czech Republic*

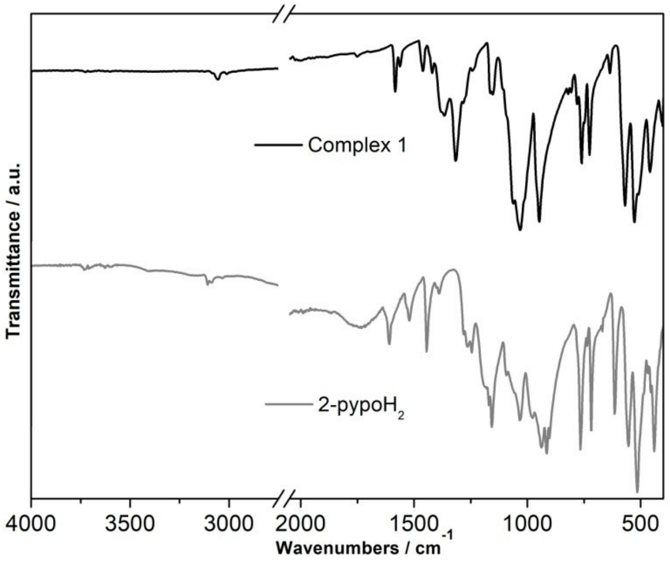

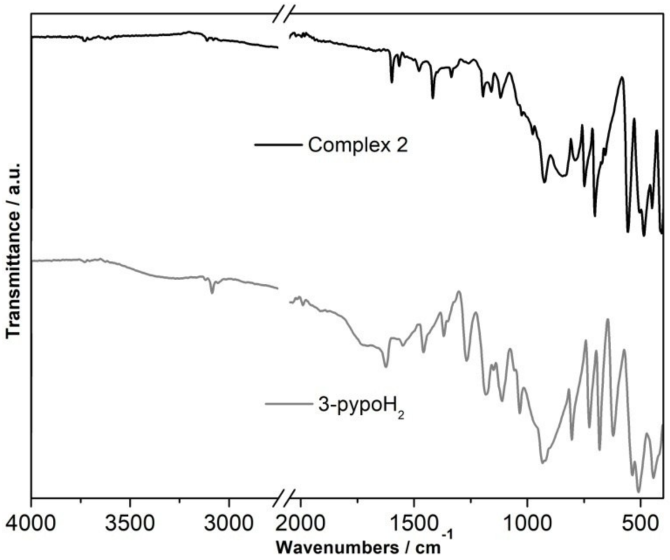

Fig. S1 IR spectra of ligands 2-pypoH_2 and 3-pypoH_2 and their complexes **1** and **2.**

Table S1 Selected bond lengths [Å] and angles [°] for complex **1**.

Ag(1)-O(5)#1	2.272(2)
Ag(1)-O(4)#2	2.317(2)
Ag(1)-O(1)	2.337(3)
Ag(1)-O(4)	2.421(2)
Ag(2)-O(5)#2	2.334(2)

Ag(2)-O(3)	2.382(2)
Ag(2)-O(1)	2.393(2)
Ag(2)-O(8)#4	2.421(2)
Ag(3)-O(2)	2.327(2)
Ag(3)-O(3)#5	2.328(2)
Ag(3)-O(6)	2.436(2)
Ag(4)-N(1)	2.212(3)
Ag(4)-O(6)	2.234(2)
Ag(4)-O(3)	2.445(2)
Ag(4)-O(4)	2.532(2)
Ag(5)-O(7)	2.294(2)
Ag(5)-O(9)	2.336(2)
Ag(5)-O(8)	2.430(2)
Ag(5)-O(2)#4	2.491(2)
Ag(6)-N(2)#6	2.237(3)
Ag(6)-O(9)#4	2.323(2)
Ag(6)-O(7)	2.434(2)
Ag(6)-O(8)	2.629(3)
Ag(6)-O(10)#5	2.658(3)
Ag(7)-N(4)	2.284(3)
Ag(7)-O(9)#6	2.461(2)
Ag(7)-O(10)	2.471(3)
Ag(7)-O(2)#3	2.485(2)
Ag(7)-O(7)	2.661(3)
N(3)-O(10)	1.210(5)
N(3)-O(11)	1.214(4)
N(3)-O(12A)	1.303(6)
N(3)-O(12B)	1.367(10)
O(1)-P(3)	1.514(3)
O(2)-P(1)	1.522(2)
O(3)-P(1)	1.526(2)
O(4)-P(2)	1.522(2)
O(5)-P(2)	1.516(3)
O(6)-P(2)#5	1.530(2)
O(7)-P(1)	1.522(2)
O(8)-P(3)#6	1.522(3)
O(9)-P(3)	1.538(2)

O(5)#1-Ag(1)-O(4)#2	131.45(9)
O(5)#1-Ag(1)-O(1)	106.59(9)
O(4)#2-Ag(1)-O(1)	107.25(9)
O(5)#1-Ag(1)-O(4)	124.13(9)
O(4)#2-Ag(1)-O(4)	80.56(9)
O(1)-Ag(1)-O(4)	102.22(9)
O(5)#2-Ag(2)-O(3)	153.12(8)
O(5)#2-Ag(2)-O(1)	102.65(9)
O(3)-Ag(2)-O(1)	78.46(9)
O(5)#2-Ag(2)-O(8)#4	100.06(8)
O(3)-Ag(2)-O(8)#4	104.38(8)
O(1)-Ag(2)-O(8)#4	111.97(9)
O(2)-Ag(3)-O(3)#5	120.80(8)
O(2)-Ag(3)-O(6)	93.48(8)
O(3)#5-Ag(3)-O(6)	127.78(8)
N(1)-Ag(4)-O(6)	153.01(10)
N(1)-Ag(4)-O(3)	125.25(9)
O(6)-Ag(4)-O(3)	81.46(8)
N(1)-Ag(4)-O(4)	78.57(9)
O(6)-Ag(4)-O(4)	108.41(8)
O(3)-Ag(4)-O(4)	89.50(8)
O(7)-Ag(5)-O(9)	123.81(8)
O(7)-Ag(5)-O(8)	83.40(8)
O(9)-Ag(5)-O(8)	150.03(8)
O(7)-Ag(5)-O(2)#4	152.95(8)
O(9)-Ag(5)-O(2)#4	78.57(8)
O(8)-Ag(5)-O(2)#4	79.76(8)
N(2)#6-Ag(6)-O(9)#4	151.17(10)
N(2)#6-Ag(6)-O(7)	117.23(10)
O(9)#4-Ag(6)-O(7)	91.40(8)
N(4)-Ag(7)-O(9)#6	158.89(10)
N(4)-Ag(7)-O(10)	112.97(11)
O(9)#6-Ag(7)-O(10)	79.41(9)
N(4)-Ag(7)-O(2)#3	115.06(10)
O(9)#6-Ag(7)-O(2)#3	76.41(8)
O(10)-Ag(7)-O(2)#3	102.82(11)
O(10)-N(3)-O(11)	127.2(4)
O(10)-N(3)-O(12A)	116.9(4)

O(11)-N(3)-O(12A)	111.6(4)
O(10)-N(3)-O(12B)	97.9(5)
O(11)-N(3)-O(12B)	113.4(5)
O(12A)-N(3)-O(12B)	75.8(5)

Symmetry transformations used to generate equivalent atoms:

#1 -x+2,-y,-z+2 #2 -x+1,-y,-z+2 #3 x+1,y,z

#4 -x,-y,-z+1 #5 x-1,y,z #6 -x+1,-y,-z+1

Table S2 Selected bond lengths [Å] and angles [°] for complex **2**.

Ag(1)-N(1)	2.1219(15)
P(1)-O(1)	1.4993(13)
P(1)-O(3)	1.5181(14)
P(1)-O(2)	1.5670(14)
O(2)-H(2)	0.760(19)
O(3)-H(3O)	1.2100
N(1)-Ag(1)-N(1)#1	180.0
O(1)-P(1)-O(3)	116.09(8)
O(1)-P(1)-O(2)	113.27(7)
O(3)-P(1)-O(2)	105.95(8)
C(5)-N(1)-Ag(1)	125.31(12)
C(1)-N(1)-Ag(1)	116.41(12)

Symmetry transformations used to generate equivalent atoms:

#1 -x+2,-y,-z

Table S3 Crystal data and structure refinement for complexes **1** and **2**.

	**1**	**2**
Empirical formula	$C_{15}H_{12}Ag_7N_4O_{12}P_3$	C_{10} $H_{11}AgN_2O_6P_2$
Formula weight	1288.29	425.02
T, K	150(2)	150(2)
Crystal system	Monoclinic	Monoclinic
Space group	$P2_1/n$	$P2_1/n$
a, Å	5.8965(2)	8.3129(8)
b, Å	31.1708(12)	6.2896(6)
c, Å	13.7850(5)	12.7052(12)
α, deg.	90	90
β, deg.	101.007(1)	93.007(4)
γ, deg.	90	90
V, $Å^3$	2487.05(16)	663.37(11)
Z	4	2
D_{calc}, g/cm^3	3.441	2.128
μ, mm^{-1}	5.661	1.790
$F(000)$	2400	420
Crystal size, mm	0.645 x 0.299 x 0.104	0.386 x 0.250 x 0.112
θ range for data collection, °	2.471 to 28.313	2.861 to 27.489
Reflections collected/independent	113416 / 6183	7704 / 1521
R_{int}	0.0886	0.0248
Data / restraints / parameters	6183 / 12 / 375	1521 / 1 / 100
S	1.074	1.063
R_1; wR_2 [I>2σ(I)]	0.0258; 0.0414	0.0197; 0.0494
R_1; wR_2 (all data)	0.0388; 0.0441	0.0220; 0.0511
Largest diff. peak/ hole, e.Å^{-3}	0.958 / -0.887	0.477 / -0.450
